# Effect of cardiometabolic risk factors on hypertension management: a cross-sectional study among 28 physician practices in the United States

**DOI:** 10.1186/1475-2840-9-7

**Published:** 2010-02-01

**Authors:** Daniel A Belletti, Christopher Zacker, Jenifer Wogen

**Affiliations:** 1 Evidence-Based Medicine, Novartis Pharmaceuticals Corporation, One Health Plaza, East Hanover, NJ 07936, USA; 2 MedMentis Consulting LLC, 145 Waughaw Rd, Towaco, NJ 07082, USA

## Abstract

**Objective:**

This cross-sectional study sought to determine the prevalence of cardiometabolic risk factor clusters (CMRFCs) and their effect on BP control among hypertensive patients from 28 US physician practices.

**Methods:**

Each participating practice identified a random sample of 150-300 adults aged ≥ 18 years diagnosed with hypertension. The primary outcome variable was BP control (BP < 140/90 mmHg for non-diabetic and <130/80 mmHg for diabetic patients). CMRFCs included hypertension in addition to obesity, dyslipidemia, and diabetes.

**Results:**

Overall, 6,527 hypertensive patients were identified for study inclusion. More than half (54.3%) were female, and mean age was 64.7 years. Almost half (48.7%) were obese (BMI ≥ 30 kg/m^2^). About 1 in every 4 patients (25.3%) had diabetes, and 60.7% had dyslipidemia. Mean blood pressure was 132.5/77.9 mmHg, and 55.0% of all patients had controlled BP; 62.4% of non-diabetic patients, and 33.3% of diabetic hypertensive patients, had BP controlled to recommended levels. Most (81.7%) hypertensive patients had ≥ 1 cardiometabolic risk factor, and 12.2% had all 3 risk factors. As compared to hypertensive patients without additional risk factors, adjusted odds ratios for BP control were significantly lower for all combinations of CMRFCs (ORs 0.15-0.83, all p < 0.04), with the exception of patients who had only dyslipidemia in addition to hypertension (OR = 1.09, p = NS). Prescriber adherence to recommended hypertension treatment guidelines for patients with diabetes, heart failure, or prior myocardial infarction was high. Although patients with risk factors were prescribed more antihypertensive medications than those without, hypertensive patients with all 3 risk factors were prescribed a mean of 2.4 antihypertensive medications compared to 1.7 for those with no risk factors; odds of BP control in these patients, however, was 0.23 [95% CI 0.19-0.29] that of patients with no other CMRFCs.

**Conclusions:**

Across 28 US practices, only 18% of hypertensive patients did not have any additional cardiometabolic risk factors. The high prevalence of CMRFCs presents a challenge to effective hypertension management.

## Background

Almost one-third of adults in the US (29.3%) had hypertension during 2003-04 [1]. Elevated blood pressure (BP) is a significant contributor to cardiovascular disease, and is associated with an increased risk of myocardial infarction (MI), stroke, renal failure, and heart failure. The seventh report of the Joint National Committee on Prevention, Detection, Evaluation, and Treatment of High Blood Pressure (JNC7) recommends that all hypertensive non-diabetic patients should be treated to BP less than 140/90 mmHg, and that all diabetic patients should be treated to BP less than 130/80 mmHg [2]. A number of distinct classes of antihypertensive medications are available for the treatment of essential hypertension, including angiotension-converting enzyme inhibitors (ACEI), angiotensin-II receptor blockers (ARB), beta blockers (BB), calcium channel blockers (CCB), alpha blockers, direct renin inhibitors, and diuretics, of which the thiazide-type are the most commonly prescribed. JNC7 recommends that a thiazide diuretic be included as part of the first-line medication regimen for most patients with hypertension, either alone or with a medication from another class [2].

Hypertension often co-exists with other metabolic and cardiovascular chronic conditions, especially diabetes, obesity, and dyslipidemia. Less than 20% of hypertension cases occur without one or more concomitant cardiovascular risk factors [3]. A cluster of ≥ 2 additional cardiovascular risk factors occurs in about half of hypertensive persons [3]. Among hypertensive persons, about 40% of coronary events in men, and 68% in women, are attributable to the co-occurrence of two or more additional risk factors [3]. Almost 1 in 3 US adults is obese [4]; obesity is associated with increased mortality, and is responsible for an estimated 111,909 excess deaths per year [5]. It is estimated that 40% of US hypertensive patients also suffer from obesity, and 15% also have diabetes [6]; in addition, 48% of hypertensive men, and 61% of hypertensive women, also have hyperlipidemia [7]. The co-occurrence of hypertension, diabetes, hyperlipidemia, and obesity has been referred to as cardiometabolic risk factor clusters (CMRFCs) [8].

Hypertension treatment guidelines for use of specific medication classes are based, in part, on the presence of specific underlying co-morbid conditions. For example, the use of an ACEI or ARB is the standard of care in patients with hypertension and diabetes or renal disease [2]. ACEI/ARB and BB are recommended for use in patients with heart failure or subsequent to a myocardial infarction [2]. Use of ACEI in combination with a thiazide diuretic is recommended in patients with cerebrovascular disease [2]. Current hypertension guidelines do not include recommendations for specific treatment regimens in obese hypertensive patients [9]. However, the use of medications from the beta blocker therapeutic class in obese patients without specific compelling indications may not be the optimal choice, as they may cause weight gain or make weight loss more difficult [10]. Clearly, the presence of CMRFCs can make the choice of a treatment regimen fairly complex.

While prospectively designed, randomized studies remain the "gold standard" for determining the efficacy and effectiveness of medications, retrospective studies can provide valuable clinical information among patients from "usual-care", non-clinical trial settings. Our study utilized a retrospective, cross-sectional design to evaluate the prevalence of cardiometabolic risk factors in the "real-world" physician practice setting, and the subsequent impact of these risk factors on BP control. For the current study, we used a subset of patients from a large, retrospective cross-sectional study of BP control and management practices across 28 US physician organizations. Specifically, we sought to determine the frequency of co-occurrence of specific cardiometabolic risk factors - diabetes, obesity, and dyslipidemia- and the impact of these clusters on BP control among a diverse cohort of patients diagnosed with hypertension across the US.

## Methods and Procedures

This analysis was part of a larger cross-sectional study conducted via chart reviews at 28 US physician practices. Study sites were located across the continental US, with a concentration in the Southern US (50%) as compared to the Western (18%), Midwestern (18%), or Northeastern (14%) census regions of the US. Requirements for site enrollment included an interest in study participation, capability to abstract required data elements, and an adult hypertensive patient population of sufficient size to allow a random sample of at least 150 patients. Study data were collected between November 2007 and September 2008 at the point-of-care via data entry by study investigators at each participating site using a secure web-based application. Investigators at participating study sites identified adult patients (≥ 18 years) with a hypertension diagnosis recorded in the patient's medical record (ICD9 code of 401.x or clinical diagnosis from chart) during the 12 months preceding the date of data collection at each site, with at least 1 year of visit history with the physician practice. Pregnant females were excluded from the study. Site investigators performed a random sample selection from the entire eligible patient population to construct a cohort of 150-300 newly- or previously-diagnosed hypertensive patients.

Information obtained for each patient included age, gender, and racial/ethnic background; information about specific risk factors, such as body mass index (BMI; calculated using inputted height and weight), smoking status, and documentation of lifestyle modification counseling (such as weight reduction, dietary changes, increased physical activity); current antihypertensive medication regimen; total number of different medications used for all chronic conditions; and physician specialty. Specific co-morbid conditions documented in the patient record were identified using corresponding ICD-9 codes and/or clinical diagnosis as documented in the patient's record; these included coronary arterial disease (CAD) without myocardial infarction (MI), MI, diabetes, hyperlipidemia, renal disease, heart failure, and cerebrovascular accident/transient ischemic attack.

BP control was the primary outcome variable, and was defined as BP < 140/90 mmHg for non-diabetic patients, and <130/80 mmHg for diabetic patients, as measured at each patient's most recent visit during the year preceding the date of data collection. If different measurement techniques were indicated (e.g. sitting, standing, or supine) resulting in multiple BP measurements on the most recent visit date, the sitting measurement was recorded for study purposes; however, if multiple measurements were recorded on the most recent visit date without distinction to the method of measurement, the mean of those values was recorded. JNC-7 classifications were also used to categorize patients based on BP as either normal BP (SBP < 120 mmHg and DBP < 80 mmHg), pre-hypertensive (SBP 120-139 mmHg or DBP 80-89 mmHg), stage I hypertension (SBP 140-159 mmHg or DBP 90-99 mmHg), or stage II hypertension (SBP > 159 mmHg or DBP > 99 mmHg) [2]. In addition, the BP measurement for the visit immediately preceding this most recent BP assessment was obtained.

This study was approved and monitored by an independent Institutional Review Board. Investigators at each site were trained on use of the data collection tool prior to beginning data collection and data entry. Specific chart abstraction instructions were provided for each data element as per the study protocol. Site investigators received a study guide that included detailed instructions about all data elements, as well as a copy of the IRB-approved study protocol.

This patient sample was a subset of a larger study population, which consisted of 8,250 hypertensive patients from 28 practice sites across the US. The current study focused on patients with hypertension and specific cardiometabolic risk factors; thus, a subset of the larger study population with complete data for all cardiometabolic risk factors (n = 6,527) was used for all analyses described here. Prior to study analyses, a comparison of the study population missing specific data elements versus those not missing each element was conducted, and analyses of patients with missing versus non-missing data showed no systematic differences.

### Statistical Methods

Univariate descriptive statistics (means and standard deviations for continuous variables, and frequency distributions for categorical variables) were calculated for all study variables. Bivariate analyses were also performed; the chi-square test was used for significance testing for categorical variables, and analysis of variance and Student's t-tests were used for continuous variables. Logistic regression was used to calculate the odds of BP control, adjusted for covariates, in the overall study population and in the subset of patients with co-morbid diabetes. Variables included in the final regression models were chosen based on results of bivariate analyses and clinical significance. Clustering within physicians and within practices was evaluated, but was determined to have little impact on odds ratio estimates obtained via logistic regression models. SPSS version 17.0 and SAS version 8.2 were used for all study analyses.

## Results

For this analysis of CMRFC, the cohort included 6,527 hypertensive patients. Demographic and clinical characteristics are included in Table 1. More than half (54.3%) of the cohort was female. Mean age overall was 64.7 years, and women were significantly older than men (p < 0.001). Mean BMI was 30.9 kg/m^2^, and almost half of study participants (48.7%) were obese (BMI ≥ 30 kg/m^2^). Patients treated by internal medicine specialists represented 46.9% of the hypertensive study population, followed by family practice physicians (36.9%) and cardiologists (15.2%). About 1 in every 4 members (25.3%) of the study cohort had diabetes, and 60.7% had dyslipidemia; 4.5% of the cohort had congestive heart failure, 14.3% had CAD without MI, and 7.6% had renal disease. Mean blood pressure at the most recent measurement was 132.5/77.9 mmHg. Overall, 55.0% of the study cohort had controlled BP; 62.4% of non-diabetic patients had BP controlled, while only 33.3% of diabetic hypertensive patients had controlled BP. Including antihypertensive medications, patients averaged 5.6 (+/-3.6) different chronic prescription medications, and, on average, were using a regimen consisting of 2.0 (+/-1.1) antihypertensive classes of medications. Diabetic patients used a mean of 2.3 (+/-1.2) classes of antihypertensive medication, compared to 1.9 (+/-1.1) for non-diabetic patients (p < 0.001). The most commonly used antihypertensive medication classes were diuretics (48.1%), ACE inhibitors (39.3%), and BB (36.7%). Men were more likely to be prescribed ACE inhibitors, and women were more likely to be prescribed ARBs or diuretics (p < 0.001, all comparisons).

**Table 1:** Study population demographic and clinical characteristics by gender

	Male (n = 2,955, 45.7%)	Female (n = 3,508, 54.3%)	Total (n = 6,527)
Age in years (mean, SD)	63.0 (13.2)	66.3 (13.9) (p < 0.001)	64.7 (13.7)

Age groups (%)		(p < 0.001)	

18-34 years	1.9%	1.2%	1.5%

35-44 years	6.6%	5.0%	5.7%

45-54 years	17.9%	14.8%	16.2%

55-64 years	27.4%	24.3%	25.7%

65-74 years	25.5%	23.9%	24.6%

>74 years	20.7%	30.9%	26.2%

Racial/ethnic background (%)		(p < 0.01)	

Caucasian	48.0%	45.6%	46.7%

African-American	11.9%	14.9%	13.5%

Hispanic	6.1%	5.5%	5.8%

Unknown/not documented	34.0%	34.0%	34.0%

BMI (kg/m^2 ^mean, SD)	31.0 (6.3)	30.7 (7.5) (p = 0.06)	30.9 (7.0)

Current smokers (%)	13.5%	9.2% (p < 0.001)	11.2%

Prescribed lifestyle modifications to control BP (%)	61.7%	59.2% (p < 0.05)	60.3%

Co-morbid cardiovascular-related conditions (%)			

Obesity (%)	49.8%	47.7% (p=NS)	48.7%

Diabetes	28.0%	23.0% (p < 0.001)	25.3%

Dyslipidemia	65.3%	56.8% (p < 0.001)	60.7%

Congestive Heart Failure	4.0%	4.9% (p = 0.081)	4.5%

CAD without MI	17.2%	11.8% (p < 0.001)	14.3%

CVA/TIA/carotid stenosis	5.2%	4.7% (p=NS)	5.0%

Myocardial Infarction	2.9%	2.1% (p = 0.039)	2.5%

Renal disease/insufficiency	7.8%	7.4% (p=NS)	7.6%

SBP- most recent measurement in mmHg (mean, SD)	132.0 (16.8)	132.8 (16.9) (p = 0.049)	132.5 (16.9)

DBP- prior measurement in mmHg (mean, SD)	78.7 (11.1)	77.2 (10.6) (p < 0.001)	77.9 (10.9)

Physician Specialty		(p = 0.027)	

Family Practice	35.9%	37.8%	36.9%

Internal Medicine	46.4%	47.3%	46.9%

Cardiology	16.6%	14.0%	15.2%

Other	1.1%	1.0%	1.0%

Total # of medications for all chronic conditions (mean, SD)	5.3 (3.4)	5.9 (3.7) (p < 0.001)	5.6 (3.6)

Total # of antihypertensive medication classes in regimen	1.9 (1.1)	2.0 (1.1) (p=NS)	2.0 (1.1)

Antihypertensive medication use by class (%)			

ACE inhibitor	44.9%	34.5% (p < 0.001)	39.3%

ARB	25.6%	29.7% (p < 0.001)	27.8%

BB	36.5%	36.7% (p=NS)	36.7%

CCB	27.6%	29.4% (p=NS)	28.6%

Any diuretic	43.4%	52.1% (p < 0.001)	48.1%

**Figure 1:**
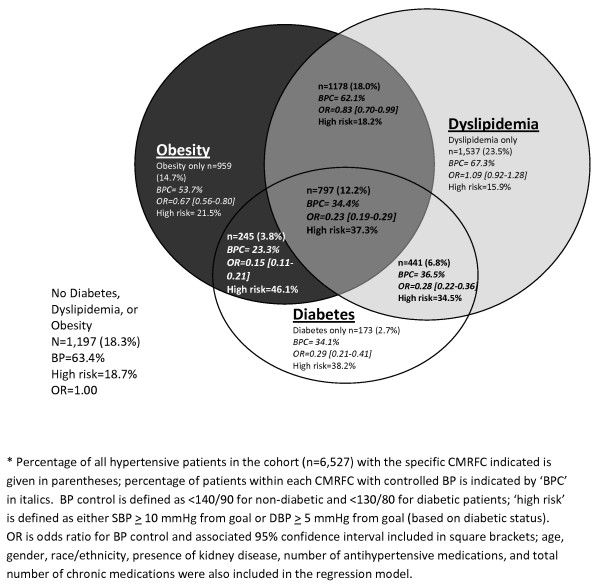
Venn diagram of cardiometabolic risk factor clusters (CMRFC) and association with BP control among hypertensive patient cohort (n = 6,527)*. Among the study hypertensive patient cohort, the co-occurrence of the cardiometabolic risk factors obesity, dyslipidemia, and diabetes are depicted. Obese hypertensive patients with diabetes had the lowest adjusted odds ratios of BP control.

Co-occurrence of cardiometabolic risk factors is depicted in Figure 1. 81.7% of the 6,527 hypertensive patients comprising the study cohort were also obese, had dyslipidemia, and/or had diabetes; only 18.3% had no concomitant risk factors. Overall, 23.5% had dyslipidemia without any other risk factors, 18.0% were obese and had dyslipidemia, 14.7% were obese only, and 12.2% had all three cardiometabolic risk factors. Only 2.7% of patients had diabetes but no other cardiometabolic risk factors. Non-diabetic patients with dyslipidemia had the highest rates of BP control among all patients with cardiometabolic risk factors. BP control rates were lowest for obese patients with diabetes (23.3%); BP control was somewhat more common among diabetic patients with dyslipidemia than for those without, regardless of obesity status. Among hypertensive patients without any other risk factors, 63.4% had controlled BP. Adjusted odds ratios for BP control by CMRFC are also shown in Figure 1. As compared to hypertensive patients without any additional cardiometabolic risk factors, those with diabetes, obesity, and dyslipidemia were less likely to have controlled BP (OR = 0.23, 95% CI 0.19-0.29). Furthermore, with the exception of patients with only dyslipidemia in addition to hypertension, all clusters were significantly less likely to have controlled BP as compared to those without any additional cardiometabolic risk factors. Patients with diabetes and obesity (46.1%) and all 3 cardiometabolic risk factors (37.3%) were the most likely to have a BP measurement that exceeded goal BP by either 10 or more mmHg systolic or 5 or more mmHg diastolic ('high risk' category in Figure 1). Patients with hypertension and dyslipidemia only (15.9%) and hypertension without any other cardiometabolic risk factors (18.3%) were the least likely to have BP measurements exceeding this threshold. While Table 1 indicated a higher prevalence of many comorbid conditions in men as compared to women, logistic regression indicated that the presence of CMRFCs, not gender, was a significant predictor of BP control (OR = 0.95, 95% CI 0.86-1.06).

Antihypertensive medication use by CMRFC is shown in Table 2. Hypertensive patients with obesity, dyslipidemia, and diabetes were prescribed the highest mean number of antihypertensive medications (2.4 +/- 1.2), while hypertensive patients without any other CMRFCs had the fewest (1.7 +/- 1.1, p < 0.001). Patients with diabetes had higher rates of ACEI or ARB usage than patients without diabetes, regardless of whether additional cardiometabolic risk factors were present. Among patients with 2 risk factors, those with diabetes and dyslipidemia who were not obese were least likely to be prescribed a diuretic, and obese diabetic patients (without dyslipidemia) were least likely to be prescribed BB (Table 2). Among all patients, use of BB did not appear to differ between obese (37.7%) and non-obese patients (35.6%, p=NS). Obese patients were prescribed a greater number of antihypertensive medications as compared to non-obese patients (2.1 +/- 1.2 vs 1.8 +/- 1.1, p < 0.001), as were diabetic as compared to non-diabetic patients (2.3 +/- 1.2 vs 1.9 +/- 1.1, p < 0.001). Patients with dyslipidemia were also prescribed more antihypertensive medications than those without (2.0 +/- 1.1 vs 1.9 +/- 1.1, p < 0.001). Among the subset of obese patients with diabetes (n = 1,042), those with controlled BP were prescribed a similar mean number of antihypertensive medication classes as those with uncontrolled BP (2.4 +/- 1.2 vs. 2.4 +/- 1.2, p = 0.46). A higher proportion of obese diabetic patients who were prescribed a regimen containing an ACEI/ARB had controlled BP than among those not using ACEI/ARB (33.3% vs 25.5%, p < 0.04), unadjusted for clinical and demographic characteristics. Among this obese diabetic patient subset, a higher proportion of patients prescribed a regimen containing a CCB had BP controlled than those not using CCB, although this difference was not statistically significant (27.7% vs vs 33.7%, p = 0.055).

**Table 2:** Cardiometabolic risk factor clusters by total number and specific classes of antihypertensive medication in drug regimen

Number of Cardiometabolic Risk Factors	Cardiometabolic Risk Factor Cluster	ACE or ARB (%)	Any Diuretic (%)	CCB (%)	BB (%)	Total # (%) of Patients	# of antihypertensive meds (mean, SD)
**0**	**-O -DM -HL**	57.6%	42.7%	25.9%	31.1%	1197 (18.3%)	1.7 (1.1)

**1**	**-O -DM +HL**	60.3%	42.3%	29.1%	36.7%	1537 (23.5%)	1.8 (1.0)

	**+O -DM -HL**	60.5%	50.6%	28.5%	31.8%	959 (14.7%)	1.9 (1.1)

	**-O +DM -HL**	67.1%	45.7%	27.2%	38.7%	173 (2.7%)	2.0 (1.1)

**2**	**+O -DM +HL**	65.1%	53.3%	26.7%	39.8%	1178 (18.0%)	2.0 (1.1)

	**-O +DM +HL**	78.7%	43.8%	29.5%	42.6%	441 (6.8%)	2.1 (1.2)

	**+O +DM -HL**	73.5%	52.2%	32.7%	36.3%	245 (3.8%)	2.2 (1.3)

**3**	**+O +DM +HL**	82.1%	58.2%	32.1%	42.0%	797 (12.2%)	2.4 (1.2)

**Total (n = 6,527)**		**65.3%**	**48.1%**	**28.5%**	**36.6%**	**6527 (100.0%)**	**2.0 (1.1)**

O = obese (BMI ≥ 30); DM = diabetes; HL = dyslipidemia.

Row percentages by medication class do not total to100% since patients may be using more than one antihypertensive medication class.

**Figure 2:**
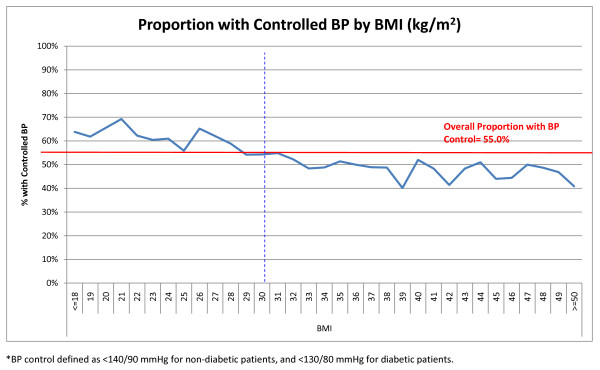
Relationship between BMI (kg/m^2^) and Proportion of Entire Study Cohort (6,527) with Controlled BP*. The proportion of hypertensive patients with controlled BP decreased with increasing patient BMI.

The relationship between BMI and BP control is depicted in Figure 2. As BMI increased in our study cohort, the proportion of patients with controlled BP decreased. The proportion of patients with controlled BP was higher for those with BMI categorized as 'normal' (<25 kg/m^2^) or 'overweight' (25-29 kg/m^2^); however, at BMI ≥ 33 kg/m^2^, the proportion with controlled BP declined, and remained lower than the overall BP control rate for the study cohort.

Use of specific antihypertensive medication classes by compelling indications is depicted in Table 3. Patients with diabetes were significantly more likely to be prescribed an ACEI or an ARB than patients without diabetes (78.3% vs 60.8%, p < 0.001), and patients with heart failure were more likely to be prescribed an ACEI, ARB, or BB than those without (88.7% vs 80.3%, p < 0.001). 88.3% of patients with a prior MI were prescribed an ACEI, ARB, or BB. However, only 67.7% of patients with renal disease were prescribed an ACEI or ARB, and only 14.6% of patients with a history of a stroke, TIA, or carotid stenosis were prescribed an ACEI in combination with a thiazide diuretic.

**Table 3:** Recommended antihypertensive medication class use among hypertensive patients with compelling indications

Antihypertensive therapies indicated for compelling indications		With Compelling Indication + Indicated Therapy	Without Compelling Indication + Indicated Therapy	p-value
**Compelling Indication**	**Indicated Therapy Regimen**	**%**	**%**	

Diabetes	Percentage of patients with HTN+DM prescribed ACEI/ARB	78.3%	60.8%	<0.001

Heart failure	Percentage of patients with HTN+HF prescribed ACEI/ARB or BB	88.7%	80.3%	<0.001

Post-MI	Percentage of patients with HTN+MI prescribed ACEI/ARB or BB	88.3%	80.5%	<0.02

Chronic kidney disease (CKD)	Percentage of patients with HTN+CKD prescribed ACEI/ARB	67.7%	65.1%	ns

Post-stroke	Percentage of patients with HTN+stroke-TIA-carotid stenosis prescribed thiazide diuretic and ACEI	14.6%	13.7%	ns

Among the subset of hypertensive patients with diabetes (n = 1,656), clinical and demographic characteristics and relationship to BP control is shown in Table 4. Only 21% of African-American diabetic patients had controlled BP. Compared to patients with uncontrolled BP, diabetic patients with controlled BP were older, had a lower mean BMI, and were using more prescriptions for all chronic conditions (8.1 vs 7.1, p < 0.001), but not more antihypertensive medications. The most common antihypertensive medications prescribed to diabetic hypertensive patients were diuretics, ACE inhibitors, and BB. Adjusted odds ratios for probability of BP control for the diabetic hypertensive patient subset are included in Table 5. Among diabetic hypertensive subjects (n = 1,656), African-Americans (OR = 0.441, 95% CI 0.312-0.623) and Hispanics (OR = 0.580, 95% CI 0.391-0.859) were significantly less likely to have controlled BP compared to Caucasian subjects. Presence of renal disease (OR = 1.703, 95% CI 1.232-2.356) and increasing total number of chronic prescription medications were both associated with an increased probability of BP control in diabetic hypertensive patients. Use of CCB was associated with a lower odds of controlled BP (OR = 0.672, 95% CI 0.471-0.958) as compared to diabetic patients prescribed regimens not containing CCB.

**Table 4:** Diabetic Cohort (n = 1,656) Clinical and Demographic Characteristics by BP Control Status

	Uncontrolled BP (n = 1,105)	Controlled BP (<130/80 mmHg) (n = 551)	Total (N = 1,656)
Age (years; mean, SD)	64.8 (12.3)	66.8 (12.0) (p < 0.01)	65.4 (12.2)

Gender- Male (n,%)	542 (65.5%)	286 (34.5%)	828 (50.6%)

Female (n,%)	549 (67.9%)	259 (32.1%) (p=NS)	808 (49.4%)

BMI (kg/m^2^; mean, SD)	33.5 (7.5)	32.7 (7.8) (p < 0.05)	33.2 (7.6)

Normal (n,%)	118 (59.9%)	79 (40.1%)	197 (11.9%)

Overweight (n,%)	276 (66.2%)	141 (33.8%)	417 (25.2%)

Obese (n,%)	521 (68.1%)	244 (31.9%)	765 (46.2%)

Morbidly obese (n,%)	190 (68.6%)	87 (31.4%)	277 (16.7%)

Racial/ethnic background (n,%)		(p < 0.001)	

Caucasian	452 (60.6%)	294 (39.4%)	746 (45.0%)

African-American	211 (78.7%)	57 (21.3%)	268 (16.2%)

Hispanic	112 (71.8%)	44 (28.2%)	156 (9.4%)

Unknown/not documented	330 (67.9%)	156 (32.1%)	486 (29.3%)

Prescribed lifestyle modifications to control BP (%)	706 (64.8%)	353 (65.0%)	1059(64.8%)

Number of antihypertensive medications (mean, SD)	2.2 (1.2)	2.3 (1.2) (p=NS)	2.3 (1.2)

Number of chronic medications (mean, SD)	7.1 (3.6)	8.1 (4.0) (p < 0.001)	7.4 (3.8)

Antihypertensive medication class	Use among uncontrolled patients (n = 1,105)	Use among controlled patients (n = 551)	Use among all patients (n = 1,656)

ACEI/ARB	75.7%	81.7% (p < 0.02)	78.3%

BB	39.7%	43.6% (p=NS)	41.0%

CCB	33.4%	26.1% (p < 0.01)	31.0%

Any diuretic	50.6%	55.4% (p=NS)	52.2%

	**% with BP control among patients with condition**	**% with BP control among patients without condition**	

Hyperlipidemia (%)	35.1%	27.8% (p = 0.006)	

Renal disease/insufficiency (%)	46.7%	31.4% (p < 0.001)	

CAD without MI (%)	38.4%	32.1% (p < 0.04)	

ACEI and/or ARB use (%)	34.7%	28.1% (p < 0.03)	

**Table 5:** Odds ratios for BP control among diabetic hypertensive study population (n = 1,656)

Variable	Levels	Odds Ratio relative to first row	95% Confidence Interval
Age		1.007	0.997, 1.017

Gender	Male		

	Female	1.102	0.886, 1.371

Race/ethnicity	Caucasian		

	African-American	0.441	0.312, 0.623

	Hispanic	0.580	0.391, 0.859

	Missing/other	0.728	0.566, 0.937

Obese		0.818	0.647, 1.036

Dyslipidemia		1.134	0.874, 1.472

Renal disease		1.703	1.232, 2.356

Number of antihypertensive medication classes	1		

	None	1.619	0.790, 3.316

	2	1.162	0.774, 1.744

	3	0.795	0.428, 1.476

	>=4	1.188	0.530, 2.662

Total number of chronic prescriptions	0-3		

	4-6	2.069	1.373, 3.117

	7-9	2.387	1.564, 3.645

	>=10	2.704	1.740, 4.202

Any ACEI/ARB use	No		

	Yes	1.343	0.921, 1.959

Any CCB use	No		

	Yes	0.672	0.471, 0.958

Any diuretic use	No		

	Yes	1.174	0.810, 1.703

Any BB use	No		

	Yes	0.962	0.687, 1.348

## Discussion

About 4 of every 5 (82%) patients in this study had 1 or more cardiometabolic risk factor, in addition to hypertension. Almost half (49%) of hypertensive subjects were also obese, and the proportion of hypertensive patients with controlled BP decreased continuously with increasing BMI. With the exception of dyslipidemia alone, the co-occurrence of combinations of CMRFCs significantly reduced the likelihood of BP control as compared to hypertensive patients without CMRFCs. Clearly diabetes, which was associated with a BP control rate of 33%, and obesity, which is interrelated with diabetes, have synergistic roles in hypertension management, as evidenced by the 23% control rate for patients with both of these cardiometabolic conditions. However, logistic regression results (Figure 1) suggest that individually, obesity and diabetes (regardless of the presence or absence of the other condition) both reduce the odds of controlled BP among hypertensive patients. Patients with more CMRFCs were prescribed a higher mean number of antihypertensive medications than those without, although the absolute difference (0.7) was not large However, odds of BP control in this patient subset was 0.23 [95% CI 0.19-0.29] that of patients with hypertension and no other CMRFCs.

A recent study by Ong et. al. using 1999-2004 National Health and Nutrition Examination Survey (NHANES) data found similar odds of BP control associated with diabetes status as the current study [7]. However, the authors found that risk factors studied were more common for women than men. Other studies using different data sources have found the opposite effect of gender [8]. Majernick et. al., in a practice-based retrospective chart review study of 631 medication-treated hypertensive patients, found that female gender, along with a higher Framingham Risk Score and presence of diabetes, were associated with reduced odds of achieving BP control [11]. In contrast, while men in our study population had a higher prevalence of diabetes, CAD, renal disease, dyslipidemia, and prior MI, adjusted odds of BP control were not different for men as compared to women.

A recent study by Kaiser Permanente found that the adjusted relative risk of cardiovascular events was highest for hypertensive patients with diabetes, hyperlipidemia, and obesity (RR = 2.80, 95% CI 2.48-3.17), and patients with all 3 comorbid conditions had the highest cumulative total medical-care costs [12]. Other research has shown that CMRFCs result in substantial annual medical expenditures in the US, even independently of the cost of cardiovascular disease [8]. Sullivan et al found that medical expenditures attributable to CMRFCs (defined as diabetes, hypertension, hyperlipidemia, and/or overweight/obesity) in the US approached $80 billion; for each individual with CMRFCs, $5477 in annual medical expenditures was attributed to CMRFCs [8]. Other work by Sullivan and colleagues has indicated that CMRFCs also have a negative impact on health-related quality of life [13] and work productivity [14]. In a population of 5,512 employees, Burton et al. demonstrated a deleterious effect on work productivity as the number of metabolic risk factors per employee increased along with a decrease in health perception [15]. Thus, CMRFCs may affect individual self-health perception, quality of life, productivity, and health care resources consumed; furthermore, the impact may become more pronounced as the number of CMRFCs in an affected individual increases.

Metabolic syndrome, defined as the co-occurrence of 3 or more of 5 risk factors (elevated waist circumference, elevated triglycerides, reduced high density lipoprotein cholesterol, elevated BP, and elevated fasting glucose), differs from the CMRFC concept in that usually CMRFCs are defined by a clinical diagnosis of hypertension, diabetes mellitus, dyslipidemia, and/or obesity (based on body mass index). The prevalence of metabolic syndrome [16], is estimated at 34% of the US adult population [17]. Metabolic syndrome is associated with an approximately doubled risk of atherosclerotic cardiovascular disease events [16]. The assessment of metabolic syndrome was outside the scope of the current study, as some components that comprise metabolic syndrome were not available for this study. Assessment of CMRFCs may be more useful and practicable in retrospective studies, as several of the components of metabolic syndrome are not usually available for chart review studies.

The presence of CMRFCs may have an impact on the specific choice of antihypertensive medication class prescribed by the physician for the hypertensive patient. Physician adherence to guidelines recommending use of an ACEI or ARB in hypertensive diabetic patients in our study was high. About one-quarter of our hypertensive patient population had diabetes, and 78% of these patients were prescribed an ACEI and/or ARB. Our findings are similar to those from other research which has estimated that 85% of diabetic hypertensive patients with renal involvement, and 71% of those without renal involvement, are prescribed an ACEI and/or ARB [18]. Both clinical trials and "real-world" studies indicate that obese hypertensive patients are more difficult to treat than their non-obese counterparts [19]. In this study, the presence of obesity did not seem to have an effect on choice of antihypertensive medication regimen. Adherence to recommended treatment regimens for hypertensive patients with the compelling indications of heart failure (89%) or history of myocardial infarction (88%) was high in our study population.

About half of our hypertensive patient population was obese, compared to about 40% of hypertensive subjects from NHANES 1999-2002 [6]. We observed a decreasing probability of BP control associated with increasing patient BMI. Obesity has been increasing in the past several decades, from 22.9% in NHANES 1988-1994 to 32.2% using NHANES 2003-2004 [20]. Framingham Study data has shown that obesity and weight gain are the most important determinants of the development of hypertension, as well as predicting that other cardiovascular risk factors will cluster with elevated BP [3]. Our study population exhibited a clear, consistent relationship between increasing BMI and BP control, but little variation in total number of antihypertensive medications used. Since the prevalence of obesity in the US is increasing among most demographic subgroups [4], therapeutic management which targets obese hypertensive patients is critical to successful BP control. The higher prevalence of obesity among hypertensive subjects in our study may be related to selection bias regarding the health-care seeking status of our study population, or may be related to the increase in obesity prevalence overall in the US that may not yet be reflected in available data sources, which reflect data from 2004. It is projected that if current obesity trends continue, by the year 2015, 75% of adults would be overweight or obese [21].

Although our study provides useful information about the prevalence of CMRFCs and their impact on hypertension management, it is important to consider several limitations. A considerable number of patients had missing data for several variables, including race/ethnicity and smoking status. Other potential factors, including duration or etiology of hypertension, duration of antihypertensive medication use, severity of concomitant conditions (such as diabetes, CHF, and CAD), prior antihypertensive medication failure, and type of health insurance coverage were not available for analysis. Other factors which were beyond the scope of the current study which may have played a role in BP control include dosing optimization of currently prescribed drug regimens, patient antihypertensive medication compliance, and adherence to diet and exercise recommendations. BP measurements were obtained retrospectively via chart review, and thus these measurements were not standardized, nor were they able to be defined prospectively. While we did not examine potential seasonal effects on blood pressure, we do not expect that such an effect would impact specific patient CMRFCs differently or substantially alter study results. The cross-sectional study design employed does not permit the determination of a cause-and-effect relationship between BP control and medication use. While our hypertensive population was geographically distributed across all regions of the US, it is not representative of the US hypertensive population as a whole. Furthermore, some of the discrepancies when comparing our results to NHANES results may, in part, be due to the fact that our patient sample is a healthcare-seeking population, as participating patients were all receiving care from a physician to manage hypertension, in contrast to data from sources such as NHANES, which, while weighted to be representative of the US demographically, includes persons not receiving routine medical care, as well as undiagnosed hypertensive individuals.

## Conclusions

Our study design enabled us to assess the co-occurrence of cardiometabolic risk factors in a large hypertensive study population representing a "real-world" setting among physician practices across the US, and to estimate the impact of specific overlapping risk factors on BP control. 82% of our hypertensive study population had one or more additional cardiometabolic risk factors: almost half were obese, and 1 in 4 had diabetes. With the exception of patients with only hypertension and dyslipidemia, patients with 1 or more CMRFC were less likely to have controlled BP. The odds of BP control for hypertensive patients with all 3 CMRFCs were 23% of the odds of control for hypertensive patients with no CMRFCs, yet, on average, patients with all 3 CMRFCs were prescribed less than 1 additional antihypertensive medication class than patients with no CMRFCs. The increasing obesity prevalence and the deleterious effect of obesity on BP control is a key public health concern. Health care providers and public health officials must consider the potential compounding effects of increased obesity on BP control in future years. The high prevalence of CMRFCs in hypertensive patients presents a challenge to the management of BP, and should be considered when selecting each patient's most appropriate antihypertensive medication regimen, with consideration given to both specific medication classes comprising the regimen, as well as the overall intensity of the therapeutic regimen (dosage strength and/or number of medications used in combination). More aggressive hypertension management strategies for obese and/or diabetic hypertensive patients are warranted to improve BP control in these difficult-to-treat patient subgroups.

## Competing interests

DB and CZ are employees of Novartis, the organization which funded this study, and own stock and stock options in Novartis. JW received compensation from Novartis for her role in the conduct of the study and manuscript preparation.

## Authors' contributions

DB and CZ contributed to conception and design of the study, interpretation of data, and critical revision of the intellectual content of the manuscript. JW contributed to study design, data acquisition, analysis and interpretation of data, and drafting and critical revision of the manuscript. All authors have given final approval of the version of the manuscript to be published.
